# War Conditions in the near East (I.)

**Published:** 1920-02-07

**Authors:** 


### WAR CONDITIONS IN THE NEAR EAST (I.).

BY A NAVAL SURGEON.

ON November 3, 1918, whilst stationed at Taranto, we found ourselves under orders to proceed to Salonica to join up with the British Naval Mission which was shortly leaving for the Danube.

Obtaining what khaki outfits we could at such short notice we joined a French troopship on November 5, and were soon amidst a truly cosmopolitan crowd. There were three British ladies travelling to Salonica, also two officers and a few men, but the chief complement was made up of Serbian soldiers, of whom there were 700 on board. Add to these a small number of French officers and men and a goodly number of black troops from the French colonies, and you have a comprehensive list of our fellow-passengers. One must not, however, forget one of the lower productions of Nature, viz., a melancholy looking ox, whose importance to the ship's company was soon made manifest, as he was quickly sent into the land of mystery by his rapacious masters. It was not a pleasing sight to note how the butchers carried out their gruesome task, for kindness to animals is not one of the predominant characteristics of this part of the world.

About 3 P.M. we slipped our moorings, and, in company with two other merchant ships and two foreign destroyers, we bade good-bye to the ancient city of Taranto. This old city on our starboard side presented a very pleasing sight to the eye of the artist, but from previous acquaintance with its details one knew only too well that it was really the home of insanitation and disease. On our port side lay the new town of Taranto, leading away up the hill to the British rest camp and hospital of Cimino, which later gave temporary shelter to an unending stream of officers and men proceeding to and from the different theatres of war in the Near and Far East. Below in the harbour one saw warships of Britain, France, and Italy. Soon we were standing out to sea, and, the weather being fine, we anticipated a quick and easy voyage, provided that we were fortunate enough to escape the attentions of enemy submarines, but as luck would have it, we ran into a thick wet mist, which soon penetrated to Nature's covering and forced most to seek a warmer and dryer atmosphere down below. The ship was very fairly clean, and the food passable, the Vin Rouge provided being certainly better than the water; but the sanitary department was fit only for the lower mammalia, for in this part of the world it is considered an eccentricity to attend to the necessary calls of Nature in anything but the most filthy surroundings. The quarters for the troops were most uninviting, and it seemed that the presence of the verminous races should be much more rigidly excluded in such a home of civilisation as a modern troopship; but possibly nearness of the coloured troops made matters exceptionally difficult in this respect.

Next morning we had reached the Greek islands on the West Coast, and the pure morning air proved a great refresher after the foul atmosphere of one's cabin. Going to sea in war-time was not, as many of one's elderly relations imagine, an open-air cure, and while Aunt Mary is fondly dreaming of her nephew, temporarily released from his stuffy City office, now comfortably sleeping in his bunk, with the ozone-laden breeze playing o'er his respiratory organs, the Temporary Lieutenant, R.N.V.R., is rapidly being asphyxiated in a stuffy little cabin whose only ventilation is composed of "potted air" coming through a very dusty pipe, which every now and again becomes completely choked by the garment of some conservative A.B. who believes in "fug." The sensation on waking, due to the accumulation of $CO_2$ may be intensely interesting to the physiologist, but it is decidedly discouraging to its possessor.

Most of this day was spent travelling up the Gulf of Corinth, a wonderful, highly artistic view; but one has rarely seen such barren mountains anywhere.

Later the monotony of the afternoon—what scene does not soon pall in the expectancy of war-time?—was relieved by an al-fresco entertainment given on the forecastle by several Serbian soldiers, whose peculiar Slav music with its many minor harmonies sounded strangely fascinating to the untutored ear. Little could we foresee under what strange circumstances we should hear that music again.

(*To be continued.*)

